# Survival after surgery for lung cancer among patients with autoimmune diseases

**DOI:** 10.1007/s00595-024-02917-8

**Published:** 2024-09-25

**Authors:** Masaaki Nagano, Yue Cong, Keita Nakao, Mitsuaki Kawashima, Chihiro Konoeda, Masaaki Sato

**Affiliations:** Department of Thoracic Surgery, The University of Tokyo Graduate School of Medicine, 7-3-1 Hongo, Bunkyo-Ku, Tokyo, 113-8655 Japan

**Keywords:** Thoracic surgery, Autoimmune disease, Sublobar resection

## Abstract

**Purpose:**

While patients with autoimmune diseases (ADs) are at high risk for developing specific malignancies, including lung cancer, ADs may protect against the development of cancer through increased immune cell activity in tumors. This study aimed to investigate whether the presence of ADs affects surgical outcomes and survival after surgery for lung cancer.

**Methods:**

The medical records of 1236 patients who underwent surgery for non-small cell lung cancer between 2007 and 2018 were retrospectively reviewed. Perioperative and long-term outcomes were compared between patients with and without ADs using propensity score matching.

**Results:**

Among the included patients, 115 with ADs and 1121 without ADs underwent surgery. Using 1-to-1 propensity score matching, 114 pairs were selected. Although there were no significant differences in the perioperative outcomes of the two groups, the overall and relapse-free survival rates were significantly lower in the group with ADs than in the group without ADs.

**Conclusions:**

Surgery for lung cancer can be performed without increasing the complications in patients with ADs. However, the long-term outcomes were significantly worse in patients with ADs than in those without ADs, suggesting that close follow-up for lung cancer and careful whole-body examination might be needed for patients with ADs.

## Introduction

Autoimmune diseases (ADs) occur when immunological tolerance to autoreactive immune cells is disrupted under certain conditions, leading to an attack on an individual’s immune system by self-molecules [1]. Autoreactive immune cells promote the secretion of high-affinity autoantibodies against self-molecules and induce focal organ damage [2, 3]. An aberrant immune function and the use of immunosuppressive therapy can lead to the development of several malignancies, including lung cancer [3–5]. Several studies have indicated that patients with ADs have an increased risk of developing cancer [3, 6], and a large study found that 13.5% of patients with lung cancer had ADs before or after their cancer diagnosis [7]. In addition, a retrospective study showed that patients with lung cancer and connective tissue disease had higher mortality rates than patients with lung cancer without ADs [8]. Other studies have also reported that the survival of patients with lung cancer and ADs, including sarcoidosis, Crohn’s disease, and ulcerative colitis, is worse than that of patients without ADs [9, 10].

In contrast, it is well known that increased immune cell activity within tumors is associated with improved prognosis in many types of cancers [11]. Autoantibodies can promote immune cell activity and induce complement-mediated or antibody-dependent cytotoxicity in tumor cells [12]. Consequently, patients with ADs can have increased anti-tumor immune effects, potentially leading to better survival. For example, epidemiological studies have revealed that systemic lupus erythematosus (SLE) plays a protective role in patients with prostate, colon, breast, endometrial, and ovarian cancers and melanoma [13, 14]. Several studies have also shown that patients with lung cancer and autoimmune diseases, including SLE, rheumatoid arthritis (RA), Crohn’s disease, and ulcerative colitis, have superior survival rates to patients without ADs [15, 16].

Autoimmunity and cancer seem to be two sides of the same coin [17]. While some studies have suggested that ADs can negatively affect cancer-related outcomes, others have not substantiated these observations [18, 19]. Furthermore, no study has analyzed how ADs affect the outcomes of patients undergoing surgery for lung cancer. We aimed to investigate whether the presence of ADs affects surgical outcomes and survival after surgery for lung cancer, using a propensity score matching analysis.

## Patients and methods

This retrospective study was approved by the Ethics Committee of the University of Tokyo Hospital (Clinical Pilot Study No. 2406).

### Patients

We included patients who underwent surgery for non-small cell lung cancer (NSCLC) at the University of Tokyo Hospital from January 2007 to December 2018. We excluded patients who underwent chemotherapy or radiation preoperatively and those who underwent other surgeries simultaneously, such as gastrectomy and coronary artery bypass grafting.

### Data analyses

The following factors were evaluated for each patient: age, sex, year of surgery, height, body weight, body mass index (BMI), smoking history, Brinkman index, side and lobe of lung cancer, respiratory function, surgical approach, type of surgery, extent of lymph node dissection, histopathology of lung cancer, pathological staging, duration of operation, intraoperative blood loss, length of postoperative stay, surgical complications, adjuvant chemotherapy, and history of ADs at the time of lung cancer surgery. Staging was based on the seventh edition of the TNM classification of malignant tumors. Histological diagnoses were made based on the World Health Organization (WHO) classification [20]. ADs were classified into 1 of the following categories [21]: multiple (RA, systemic sclerosis, sarcoidosis, Sjögren’s syndrome, SLE, IgG4 related disease, Behçet’s disease, adult Still’s disease), endocrine (Hashimoto’s disease, type I diabetes, Graves’ disease, Addison’s disease, lymphocytic hypophysitis), gastrointestinal (primary biliary cirrhosis, ulcerative colitis, autoimmune hepatitis), cutaneous (polymyalgia rheumatic, polymyositis/dermatomyositis), cardiovascular (Takayasu arteritis, microscopic polyangitis, granulomatosis with polyangitis, eosinophilic granulomatosis with polyangitis), and hematopoietic (autoimmune hemolytic anemia, immune thrombocytopenic purpura, antiphospholipid syndrome). Patients with asthma and hyperthyroidism/hypothyroidism, with no evidence of autoimmune thyroiditis, were excluded.

### Outcome measures

Perioperative outcomes included postoperative length of hospital stay, duration of operation, intraoperative blood loss, and surgical complications. Surgical complications included reoperation, prolonged air leak (≥ 7 days), pneumonia, cardiovascular events, empyema, bronchopulmonary fistula, and others. Postoperative follow-up procedures, including chest computed tomography (CT) every 6 months, were performed for at least 5 years after surgical resection. Fluodeoxyglucose-positron emission tomography and brain magnetic resonance imaging were performed when recurrence was suspected based on the CT findings or symptoms. Recurrence-free survival (RFS) was defined as the time from the date of surgery until the date of recurrence, death, or last follow-up visit. Overall survival (OS) was defined as the time from the date of surgery until the date of death due to any cause or until the last follow-up visit. The primary outcome of this analysis was the OS rate, and the secondary outcomes were the RFS rate and perioperative outcomes (complication rate, postoperative length of hospital stay, operative time, and intraoperative blood loss).

### Statistical analysis

The patients were grouped according to the presence or absence of ADs. Patients with one AD at the time of lung cancer surgery were classified into the AD group. Background variables were likely to be present in patients with and without ADs, as this study was not a prospective randomized controlled trial. Thus, we performed propensity score matching between the groups with and without ADs to adjust for confounding factors. Propensity scores were calculated using a logistic regression model based on the following factors: age, sex, year of surgery, height, body weight, BMI, smoking history, Brinkman index, lung cancer side and lobe, respiratory function, surgical approach, type of surgery, extent of lymph node dissection, lung cancer histopathology, and pathological staging. The C-statistic was calculated to evaluate the goodness of fit. Each patient with ADs was matched with a patient without ADs who had the closest estimated propensity score within a specified range (< 0.2 of the pooled standard deviation of the estimated logits). To describe patient demographic data, categorical variables were summarized as numbers and proportions, and continuous variables were summarized as medians and interquartile ranges (IQR). Categorical variables were compared using Pearson’s chi-square test before matching and McNemar’s test after matching. Continuous variables were analyzed using the nonparametric Mann–Whitney U test before matching and paired t-test after matching. Survival data were calculated using the Kaplan–Meier method and compared using the stratified log-rank test. Statistical significance was set at *P* < 0.05. All statistical analyses were performed using EZR, a modified version of R commander that adds statistical functions frequently used in biostatistics [22].

## Results

We identified 1236 patients who underwent surgery for NSCLC at our hospital between 2007 and 2018. Among them, 115 patients had ADs, including RA (n = 25), Hashimoto’s disease (n = 14), systemic sclerosis (n = 11), and sarcoidosis (n = 7) (Table 1). Immunosuppressive therapy with steroids, tacrolimus, methotrexate, cyclosporine, and azathioprine was administered to 48 patients with ADs (42%) at the time of surgery. In addition, 25 (22%) and 27 (25%) patients had interstitial pneumonia and a history of cancer within 5 years before surgery, respectively. The eligible patients were divided into a group without ADs (1121 patients) and a group with ADs (n = 115). Table 2 shows the patients’ characteristics, demonstrating that the proportion of female patients was significantly higher in the AD group. Height, body weight, and respiratory function (forced expiratory volume and forced vital capacity) were lower in the AD group than in the non-AD group. Age, smoking status, tumor location, surgical approach and procedures, NSCLC histopathology, and pathological staging did not differ between the two groups to a statistically significant extent. Using 1-to-1 propensity score matching, 114 pairs were selected. The C-statistic before matching was 0.679 and the distribution of patient backgrounds was closely balanced between the two groups after matching (Table 2).

**Table 1:** Autoimmune diseases (n = 115)

Disease	N
Multiple	
Rheumatoid arthritis	25
Systemic sclerosis	11
Sarcoidosis	7
Sjögren’s syndrome	5
Systemic lupus erythematosus	5
IgG4 related disease	3
Behçet’s disease	3
Adult Still’s disease	2
Endocrine	
Hashimoto’s disease	14
Type I diabetes	4
Graves’ disease	3
Addison’s disease	1
Lymphocytic hypophysitis	1
Gastrointestinal	
Primary biliary cirrhosis	5
Ulcerative colitis	2
Autoimmune hepatitis	1
Cutaneous	
Psoriasis	5
Pemphigoid	1
Pemphigus	1
Musculoskeletal	
Polymyalgia rheumatic	5
Polymyositis/dermatomyositis	4
Cardiovascular	
Takayasu arteritis	5
Microscopic polyangitis	3
Granulomatosis with polyangitis	1
Eosinophilic granulomatosis with polyangitis	1
Hematopoietic	
Autoimmune hemolytic anemia	1
Immune thrombocytopenic purpura	1
Antiphosholipid syndrome	1

**Table 2:** Characteristics of patients with or without autoimmune diseases

	Full sample				Matched			
	without ADs (n = 1121)	with ADs (n = 115)	*P* value	Standardized mean difference	without ADs (n = 114)	with ADs (n = 114)	*P* value	Standardized mean difference
Age (years), median	70	70	0.72	0.002	70	70	0.86	0.026
Sex								
Male, n (%)	682 (61)	52 (45)	0.001	0.32	50 (44)	51 (45)	1	0.018
Female, n (%)	439 (39)	63 (55)			64 (56)	63 (55)		
Year								
2007–2010, n (%)	335 (30)	24 (21)	0.03	0.26	25 (22)	24 (21)	0.39	0.023
2011–2014, n (%)	371 (33)	51 (44)			38 (33)	50 (44)		
2015–2018, n (%)	415 (37)	40 (35)			51 (45)	40 (35)		
Height (cm), median	161	159	0.05	0.19	158	159	0.45	0.096
Body weight (kg), median	58	55	0.009	0.23	54	55	0.72	0.045
BMI, median	22	22	0.1	0.15	22	22	0.95	0.009
Smoking								
None, n (%)	406 (36)	40 (35)	0.82	0.06	41 (36)	40 (35)	0.93	0.064
Past, n (%)	470 (42)	47 (41)			49 (43)	47 (41)		
Current, n (%)	245 (22)	28 (24)			24 (21)	27 (24)		
Brinkman index, median	444	500	0.57	0.094	430	500	0.73	0.046
Side								
Right, n (%)	669 (60)	62 (54)	0.23	0.12	55 (48)	61 (54)	0.49	0.10
Left, n (%)	452 (40)	53 (46)			59 (52)	53 (47)		
Lobe								
Upper, n (%)	660 (59)	55 (48)	0.20	0.253	69 (61)	55 (57)	0.21	0.025
Middle, n (%)	75 (7)	9 (8)			7 (6)	9 (8)		
Lower, n (%)	386 (34)	50 (44)			38 (33)	50 (44)		
Respiratory function								
FEV_1_ (mL), median	2190	2000	0.002	0.3	2064	2000	0.45	0.088
FEV_1_/FVC ratio (%), median	73	74	0.67	0.082	75	74	0.36	0.093
FEV_1_ (% predicted), median	98	95	0.112	0.17	97	96	0.35	0.093
FVC (mL), median	3040	2830	< 0.001	0.41	2750	2830	0.95	0.007
FVC (% predicted), median	103	101	0.03	0.26	97	101	1.0	0.001
Surgical approach								
VATS, n (%)	808 (72)	80 (70)	0.59	0.055	79 (69)	80 (70)	1	0.019
Open, n (%)	313 (28)	35 (30)			35 (31)	34 (30)		
Operation								
Wedge, n (%)	174 (16)	23 (20)	0.13	0.21	21 (18)	23 (20)	0.80	0.10
Segmentectomy, n (%)	77 (7)	10 (9)			9 (8)	10 (9)		
Lobectomy, n (%)	853 (76)	79 (69)			83 (73)	79 (69)		
Bilobectomy, n (%)	13 (1)	1 (1)			1 (1)	1 (1)		
Pneumonectomy, n (%)	4 (0.4)	2 (2)			0	1 (1)		
Lymph node dissection								
ND0, n (%)	173 (15)	24 (21)	0.14	0.22	21 (18)	24 (21)	0.87	0.074
ND1, n (%)	127 (11)	18 (16)			20 (18)	18 (16)		
ND2, n (%)	819 (73)	73 (63)			73 (64)	72 (62)		
Histopathology								
Adenocarcinoma, n (%)	870 (78)	82 (72)	0.25	0.15	83 (73)	82 (72)	0.62	0.10
Squamous, n (%)	202 (18)	26 (23)			22 (19)	26 (23)		
Others, n (%)	49 (4)	7 (6)			9 (8)	6 (5)		
TNM staging								
0, n (%)	31 (3)	4 (4)	0.52	0.22	6 (5)	4 (4)	0.65	0.013
IA, n (%)	588 (53)	50 (44)			46 (40)	50 (44)		
IB, n (%)	258 (23)	30 (26)			28 (25)	30 (26)		
IIA, n (%)	77 (7)	9 (8)			10 (9)	9 (8)		
IIB, n (%)	64 (6)	10 (9)			10 (9)	9 (8)		
IIIA, n (%)	98 (9)	12 (11)			14 (12)	12 (11)		
IIIB, n (%)	5 (0.4)	0			0	0		

*ADs* autoimmune diseases, *BMI* body mass index, *FEV* forced expiratory volume, *FVC* forced volume capacity, *IQR* inter-quartile range, *ND* lymph node dissection, *VATS* video-assisted thoracic surgery

Table 3 presents the perioperative outcomes of the propensity-score-matched groups. The postoperative length of hospital stay, operative time, and intraoperative blood loss of the two groups did not differ to a statistically significant extent. Complications were observed in 24 patients in each group (both 21%). Adjuvant chemotherapy was administered to 20 patients (18%) without ADs and 15 patients (14%) with ADs.

**Table 3:** Perioperative outcomes

	without ADs (n = 114)	with ADs (n = 114)	P value
Postoperative hospital stay (days), median (IQR)	7 (6–11)	8 (6–11)	0.35
Operation time (min), median (IQR)	188 (140–245)	196 (133–256)	0.43
Blood loss (mL), median (IQR)	100 (20–173)	90 (10–160)	0.37
Complication			
Any complication, n (%)	24 (21)	24 (21)	1
Re-operation, n (%)	5 (4)	5 (4)	1
Cardiovascular, n (%)	6 (5)	5 (4)	1
Bronchopulmonary fistula, n (%)	1 (1)	1 (1)	1
Pneumonitis, n (%)	5 (4)	11 (10)	0.21
Empyema, n (%)	3 (3)	3 (3)	1
Prolonged air leak, n (%)	9 (8)	8 (7)	1
Others, n (%)	7 (6)	10 (9)	0.63
Adjuvant chemotherapy			
Nothing, n (%)	94 (82)	99 (87)	0.75
Oral, n (%)	8 (7)	4 (4)	
Cisplatin based, n (%)	12 (11)	11 (10)	

*ADs* autoimmune diseases, *IQR* inter-quartile range

**Fig. 1:**
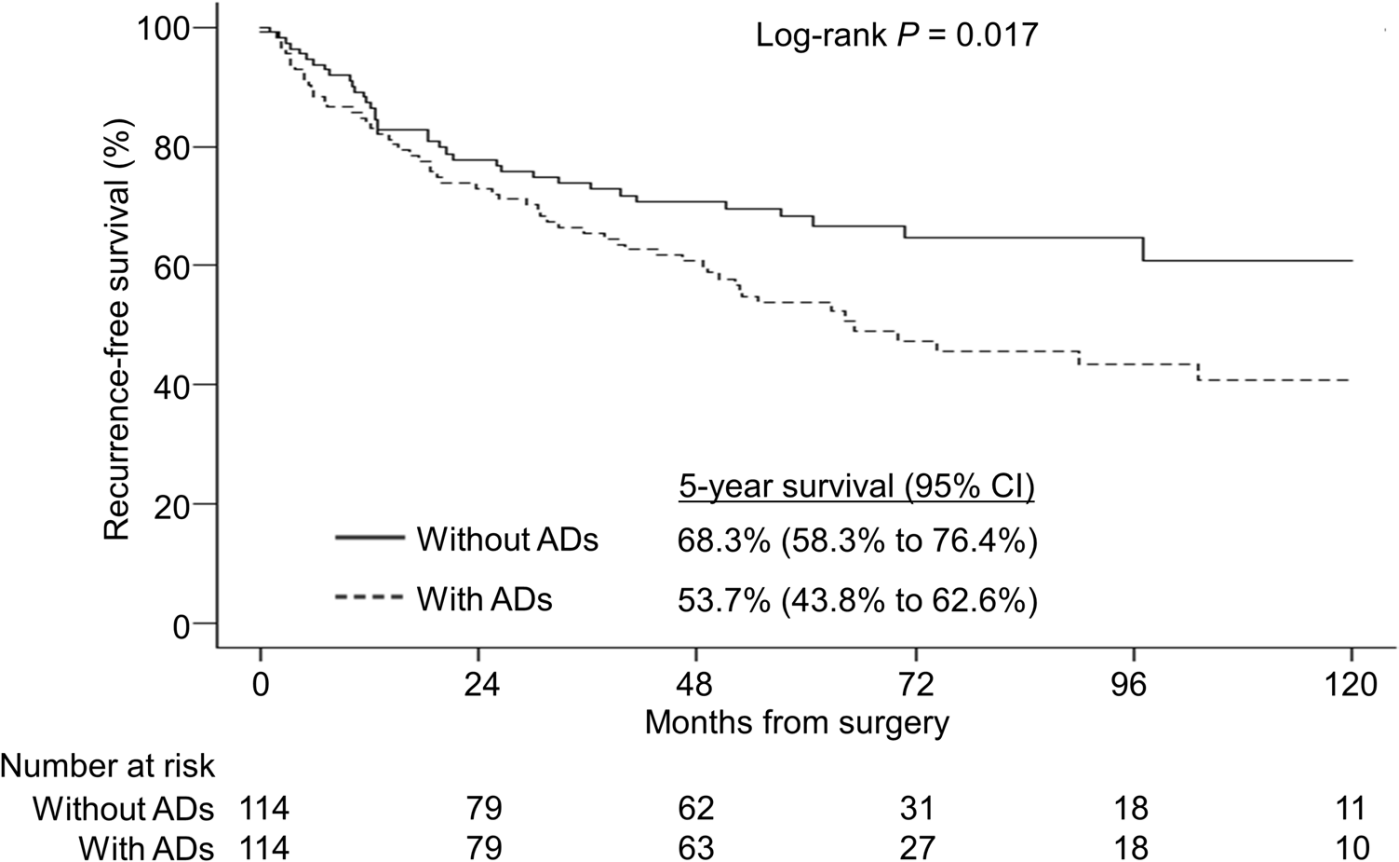
Kaplan–Meier curves of recurrence-free survival (RFS) of patients with (dotted line) and without (solid line) ADs after propensity score matching. The RFS was shorter in patients with ADs than in patients without ADs (log-rank test, *P* = 0.017). *ADs* autoimmune diseases, *CI* confidence interval

**Fig. 2:**
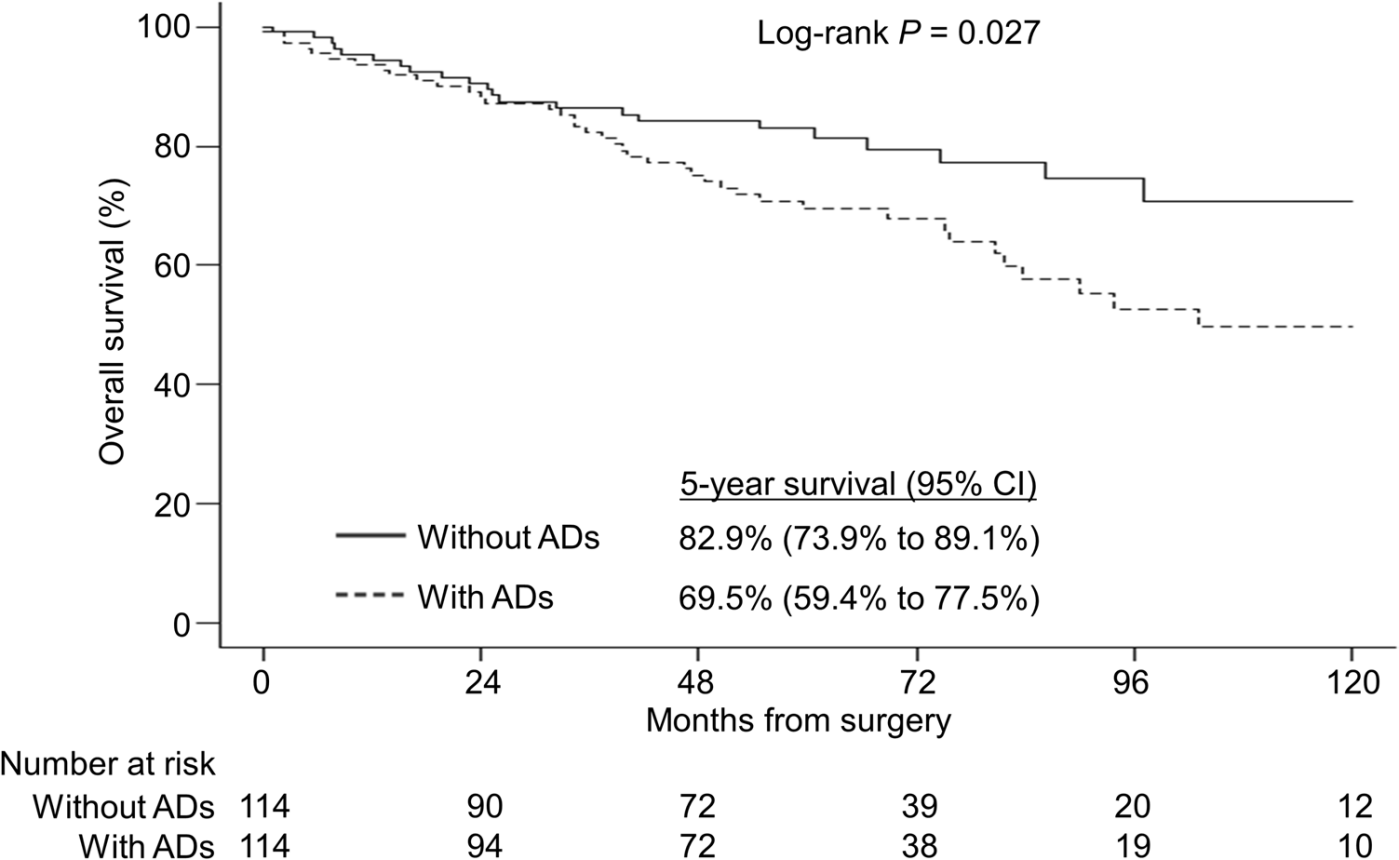
Kaplan–Meier curves of overall survival (OS) of patients with (dotted line) and patients without (solid line) ADs after propensity score matching. The OS was shorter in patients with ADs than in patients without ADs (log-rank test, *P* = 0.027). *ADs* autoimmune diseases, *CI* confidence interval

The median follow-up time was 61.1 months in the group without ADs and 60.1 months in the group with ADs. The 5-year recurrence-free survival (RFS) rates were 54% and 68% in the groups with and without ADs, respectively. The log-rank test showed a significant difference between the two groups (*P* = 0.017) (Fig. 1). After recurrence, 26% (10 39) of patients with ADs had the best supportive care without any treatment, while only 10% (3 29) of patients without ADs had the best supportive care without any treatment. Figure 2 shows the overall survival (OS) curves of the two groups. The 5-year OS rates in the groups with and without ADs were 70% and 83%, respectively. Kaplan–Meier curves showed that survival was less favorable in the group with ADs relative to the group without ADs (log-rank test, *P* = 0.027). During the follow-up period, 40 AD patients died. Causes of death included lung cancer (n = 21), pneumonia (n = 6), and other malignant diseases (n = 6). Other malignant diseases included gastric cancer (n = 3), lymphoma (n = 1), colon cancer (n = 1), and hepatocellular carcinoma (n = 1). In contrast, 23 patients without ADs died during the follow-up. The causes of death included lung cancer (n = 14), other malignant diseases (n = 3), pneumonia (n = 2), and others (n = 4).

## Discussion

The surgical outcomes of the groups with and without ADs did not differ to a statistically significant extent. However, RFS and OS rates in the group with ADs were significantly lower than those in the group without ADs, suggesting that the presence of ADs may be negatively associated with survival after surgery for NSCLC.

In all cases, before matching, there was a female predominance in the group with ADs, and patients with ADs tended to be physically smaller than patients without ADs. The majority of patients with ADs, such as SLE, systemic sclerosis, and Hashimoto’s disease, are female [23], and females are generally physically smaller than males. These facts explain why the AD group in the present study had a smaller body size and a decreased respiratory function.

With the exception of sex and physique, there were no differences in patient characteristics between the two groups before matching. Although a previous study reported that patients with ADs would receive close follow-up care for their background diseases and thus have earlier stage NSCLC [16], the results of our study do not support this assertion. In addition, our study showed that among patients with ADs, 70% and 63% of patients underwent lobectomy and mediastinal lymph node dissection, respectively. The results were not significantly different from those of the patients without ADs (76% and 73%, respectively). While limited resection, especially wedge resection, tends to be selected in poor-risk patients with ADs to prevent complications [19], many patients with ADs can undergo radical surgery as a standard-of-care treatment in our institution.

We demonstrated that among patients with ADs, surgical outcomes, including operation time, blood loss, length of postoperative hospital stay, and complications, were similar to those of patients without ADs. Several studies have shown that interstitial pneumonia and immunosuppressive treatment lead to an increased rate of complications after lung cancer [24, 25]. As some patients with ADs have interstitial pneumonia and receive immunosuppressive treatment, the surgical outcomes of patients with ADs are expected to be worse than those of patients without ADs. However, the perioperative outcomes of the two groups were similar, suggesting that surgery for patients with lung cancer and ADs could be performed safely without increasing the risk of complications. To the best of our knowledge, this is the first study to show how ADs affect morbidity and mortality in patients undergoing surgery for lung cancer. We believe that it is of great importance to compare the perioperative outcomes between patients with and without ADs, because no such studies have been reported in the relevant literature.

Long-term survival, including RFS and OS, was significantly worse in the AD group than that in the non-AD group. These results can be attributed to three factors. First, immunosuppressive therapy for ADs may promote lung cancer relapse. Although the rate of adjuvant chemotherapy in our cohort did not differ between the groups with and without ADs, the RFS was higher in the group without ADs. This result indicates that immunosuppressive conditions may facilitate lung cancer progression in patients with ADs. Dedousis et al. showed an improvement in OS and cancer-specific mortality in patients with ADs relative to those without ADs, suggesting that patients with ADs may have increased antitumor immune effects in comparison to the general population [16]. However, our study indicates that the negative effects of immunosuppressants have a greater impact on the prognosis of lung cancer after surgery than the antitumor effects due to enhanced immune activity in patients with ADs. Second, patients with ADs may not receive the standard-of-care treatment after recurrence. A large retrospective study showed that only 70% of patients with ADs had a standard of care, whereas 97% of those without ADs received this [19]. Our study showed that 26% of patients with ADs had the best supportive care without treatment after recurrence, whereas only 10% of patients without ADs had the best supportive care without treatment. These similar results may indicate that clinicians decide on more conservative treatment approaches for patients with ADs than for those without ADs. Third, the other reasons for worsened long-term outcomes in the AD group were pneumonia and malignancy in other organs. In our study, the number of patients who died of pneumonia and other malignant diseases was high in the AD group, indicating that tissue and cellular damage caused by ADs may have negative effects on long-term outcomes in patients with ADs. Previous studies showed that other malignant diseases can develop in patients with ADs [3–5], which supports our results. Six patients with ADs in our study died of other malignant diseases, half of which were gastric cancers. Thus, clinicians should be particularly aware of this type of cancer. Moreover, pneumonia might sometimes be lethal in patients with ADs receiving immunosuppressive treatment or in those with interstitial pneumonia. Patients with ADs may require blood and radiological examinations more frequently than patients without ADs after surgery to facilitate the early diagnosis of pneumonia.

The present study was associated with several limitations. First, this was a non-randomized, retrospective, single-center study. Second, the ADs in this study included a wide variety of diseases, and did not focus on specific diseases. Nonetheless, the number of patients used to investigate the correlation between ADs and lung cancer in this study was relatively large in a single institution. Moreover, while several relevant studies have used public databases and were not precisely adjusted for patient background factors [6, 16], an adjusted analysis using propensity scores was performed in our study. It is noteworthy that we adjusted for patient characteristics in detail, including the respiratory function, past medical history, tumor location, and type of surgery. Third, the AD factors that affect the long-term outcomes in patients with ADs remain unclear. Tissue and cellular damage caused by ADs, such as interstitial pneumonia, hepatitis, and colitis, may play a role in the treatment strategy for patients with lung cancer. The dose and type of immunosuppressant may also play an important role in the progression of lung cancer. However, these factors were not assessed in this study. Therefore, multicenter studies focusing on patients with certain autoimmune diseases are needed to further evaluate ADs and lung cancer survival, which can reveal whether the tissue and cellular damage caused by ADs, the overall condition of the body, or the level of immunosuppression have a greater impact on the poor prognosis of lung cancer. However, our study is noteworthy in that the ADs themselves had negative effects on the long-term outcomes in patients with lung cancer and ADs after curative surgery. We believe that this study has implications in determining the treatment strategies for patients with ADs and lung cancer.

## Conclusion

Surgery for lung cancer can be performed without increasing the incidence of complications in patients with ADs. However, these patients had significantly poor long-term outcomes. Therefore, it may be necessary to take great care of not only lung cancer but also the patient’s overall health in the management of patients with ADs.

## Abbreviations

ADs: Autoimmune diseases
BMI: Body mass index
CT: Computed tomography
IQR: Interquartile range
NSCLC: Non-small cell lung cancer
OS: Overall survival
RA: Rheumatoid arthritis
RFS: Recurrence-free survival
SLE: Systemic lupus erythematous
VATS: Video-assisted thoracic surgery
